# Long-lasting neuronal desynchronization caused by coordinated reset stimulation

**DOI:** 10.1186/1471-2202-12-S1-K3

**Published:** 2011-07-18

**Authors:** Peter A Tass

**Affiliations:** 1 Institute of Neuroscience and Medicine - Neuromodulation (INM-7), Research Center Juelich, 52425 Juelich, Germany & Department of Stereotactic and Functional Neurosurgery, University Hospital, 50924 Cologne, Germany

## 

A number of brain diseases, e.g. movement disorders such as Parkinsons disease, are characterized by abnormal neuronal synchronization. Within the last years permanent high-frequency (HF) deep brain stimulation became the standard therapy for medically refractory movement disorders. To overcome limitations of standard HF deep brain stimulation, we use a model based approach. To this end, we make mathematical models of affected neuronal target populations and use methods from statistical physics and nonlinear dynamics to develop mild and efficient control techniques. Along the lines of a top-down approach we test our control techniques in oscillator networks as well as neural networks. In particular, we specifically utilize dynamical self-organization principles and plasticity rules. In this way, we have developed coordinated reset (CR) stimulation, an effectively desynchronizing brain stimulation technique. The goal of CR stimulation is not only to counteract pathological synchronization on a fast time scale, but also to unlearn pathological synchrony by therapeutically reshaping neural networks. The CR theory, results from animal experiments as well as clinical applications will be presented: Animal and human data will be shown on electrical CR stimulation for the treatment of Parkinsons disease via chronically implanted depth electrodes.

Furthermore, acoustic CR stimulation for the treatment of subjective tinnitus will be explained. Subjective tinnitus is an acoustic phantom phenomenon characterized by abnormal synchronization in the central auditory system. In a multicenter proof of concept study it has been shown that acoustic CR stimulation significantly and effectively counteracts tinnitus symptoms as well as the underlying pathological neuronal synchronization processes.

